# Supplementary material for: In Vitro Effects of Waterborne Polyurethane 3D Scaffolds Containing Poly(lactic-co-glycolic acid)s of Different Lactic Acid/Glycolic Acid Ratios on the Inflammatory Response
Source: Polymers (Basel). 2023 Apr 4;15(7):1786. doi: 10.3390/polym15071786 (PMC10097270; doi:10.3390/polym15071786)
Supplement: Supplementary file 1 [file polymers-15-01786-s001.zip › polymers-2283512-supplementary.pdf]

Article

In Vitro Effects of Waterborne Polyurethane 3D Scaffolds Containing Poly(lactic-co-glycolic acid)s of Different Lactic Acid/Glycolic Acid Ratio on Inflammatory Response

Guanyu Zhang, Ao Zhen, Jinlin Chen, Bohong Du, Feng Luo, Jiehua Li*, Hong Tan*

College of Polymer Science and Engineering, State Key Laboratory of Polymer Materials Engineering, Sichuan University, Chengdu 610065, China

* Correspondence: jiehua_li@scu.edu.cn (J.L.); hongtan@scu.edu.cn (H.T.)

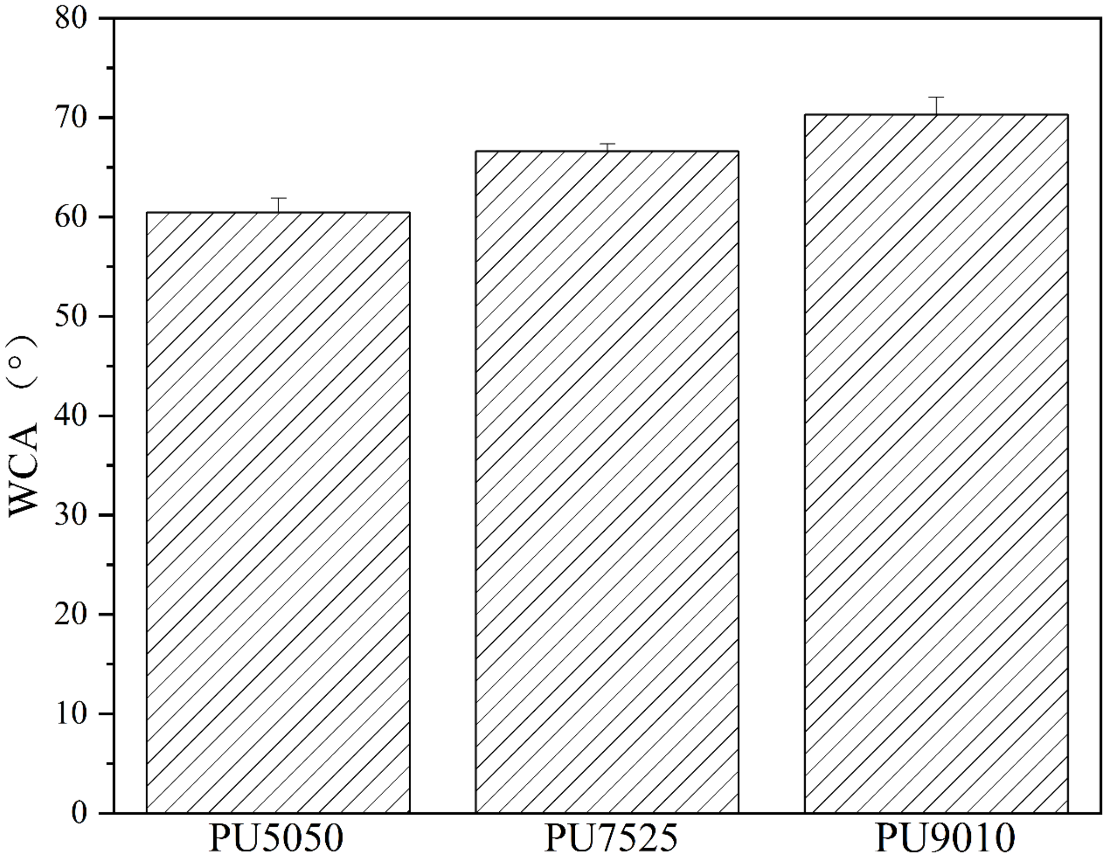

Figure S1. Water contact Angle of waterborne polyurethane films.

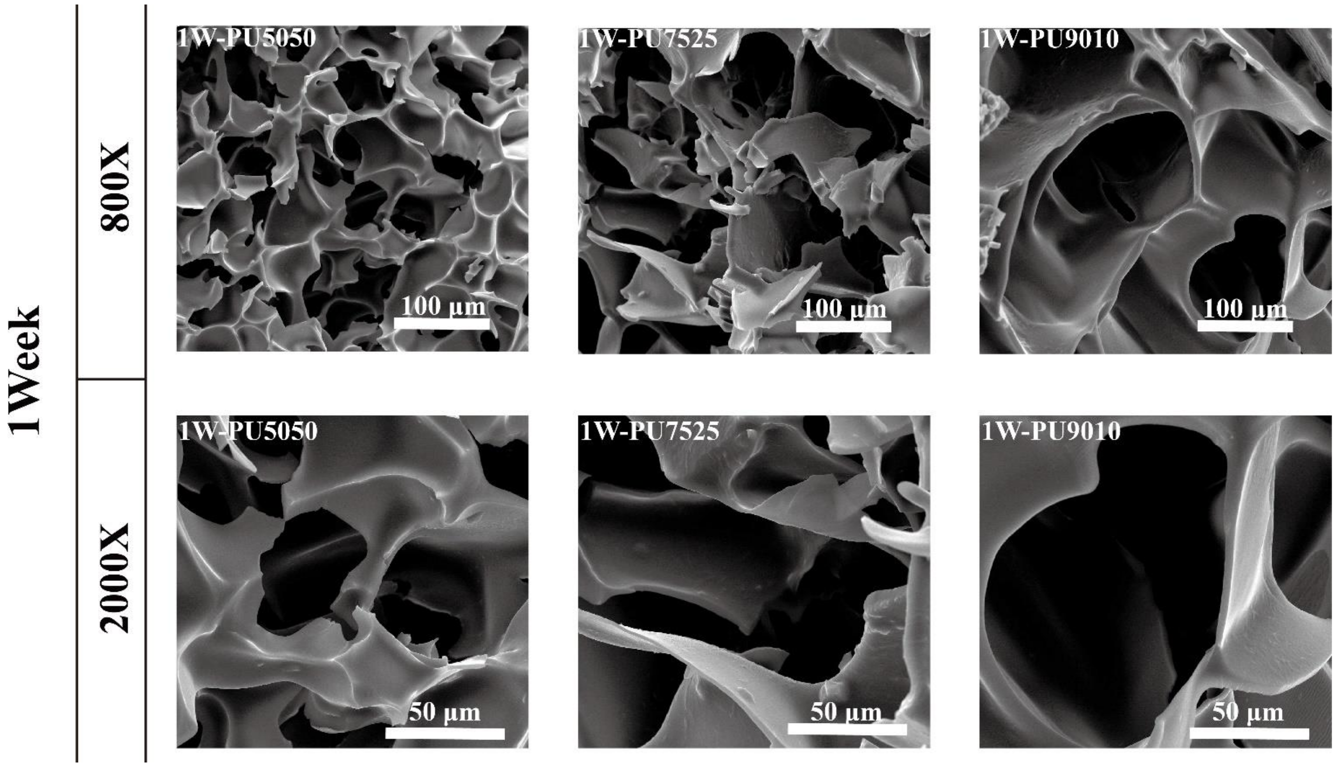

Figure S2. The WPU scaffolds' morphology of hydrolyzing 1 week.

Table S1. Details of the instruments used in this work.

Instrument	Specification	Manufacturer	Error and tolerance
Electronic balance	CP213	Ohaus Instrument Co., LTD, Shanghai	0.01 g
Electric heating constant temperature blast oven	DZG-9140A	Qixin Scientific Instrument Co., LTD., Shanghai	
Rotary evaporator	SHB-Ⅲ	Huicheng Technology & Trade Co. LTD, Zhengzhou	
Pipetting gun	Finnpipette F3	Thermo Fisher Scientific, CN	1μL
Freeze-dryer	Pilot10-15M	Biocool, Beijing	
Fourier transform infrared spectroscopy	iS50 FT-IR	NICOLET, Thermo Scientific, America	
Scanning electron microscopy	Nova Nano SEM 450	FEI Company, America	
Universal tensile tester	HZ-1004	Lixian Instrument Scientific Co., Ltd, Dongguan	0.1 N
Water contact angle	DSA100	KRUSS, China	0.1°
Cell incubator	Forma™ Steri-Cycle™ CO_2 Incubator, 184 L, Polished Stainless Steel	Thermo Fisher Scientific， CN	
Ultrapure water machine	NPTK-4G	Youpu Ultra Pure Technology Co., LTD, Sichuan	
